# Nutrient-wide associations of asthma, atopic dermatitis, and allergic rhinitis in Korean adults: a cross-sectional analysis of KNHANES 2016–2023

**DOI:** 10.1038/s41598-026-54259-9

**Published:** 2026-05-23

**Authors:** Yu Kyoung Hwang, Hyo-Jin Min

**Affiliations:** 1 Division of Allergy, Department of Internal Medicine, Chungbuk National University Hospital, 776 1 Sunhwan-ro, Seowon-gu, Cheongju-si, 28644 Chungcheongbuk-do Korea, Republic of; 2 Cheongju Osong National Advanced Clinical Trial Center, ChungBuk National University Hospital, Cheongju-si, Korea, Republic of

**Keywords:** Nutrient-wide association study, Asthma, Allergic rhinitis, Atopic dermatitis, KNHANES, Diseases, Health care, Immunology, Medical research, Risk factors

## Abstract

Diet may influence allergic disease risk through oxidative stress, inflammatory signaling, and immune pathways. However, evidence for specific nutrients remains inconsistent, particularly in Asian adult populations. We examined nutrient-wide associations with asthma, allergic rhinitis, and atopic dermatitis in a nationally representative cohort of Korean adults to systematically characterize nutrient-allergy relationships at the population level. We conducted a cross-sectional analysis of 37,808 adults (≥ 18 years) from the Korea National Health and Nutrition Examination Survey (KNHANES) 2016–2023. Physician-diagnosed asthma, allergic rhinitis, and atopic dermatitis were identified using standardized questionnaires. Dietary exposure was assessed using a 24-hour dietary recall, from which absolute daily intakes of 28 nutrients were derived. Nutrient variables were transformed as appropriate and standardized to 1-standard-deviation (SD) increments. For each outcome, we fitted survey-weighted logistic regression models with sequential adjustment for demographic and lifestyle covariates. Multiple testing was addressed using false discovery rate (FDR) control (*q* values < 0.05). In fully adjusted models, greater intakes of total dietary fiber, potassium, and magnesium were associated with lower odds of asthma and remained significant under FDR correction (*q* < 0.05), with odds ratios (OR) per 1-SD increase ranging from 0.80 to 0.90. In contrast, allergic rhinitis showed positive associations with higher intakes of total fat, vitamin E, riboflavin, and potassium, which also met the FDR criterion (ORs per 1-SD increase approximately 1.05–1.15). For atopic dermatitis, no nutrient achieved FDR significance. In this nutrient-wide evaluation among Korean adults, only a limited subset of nutrients showed robust associations with asthma or allergic rhinitis, and the direction of association varied by allergic diseases. These findings underscore heterogeneity in diet-allergy relationships and support the value of nutrient-wide approaches for prioritizing targets for future prospective research.

## Introduction

Asthma, allergic rhinitis, and atopic dermatitis are prevalent chronic inflammatory conditions that significantly impair quality of life and drive high healthcare costs worldwide, including across Asia^1^. In Korea, population-based surveys show that allergic rhinitis is particularly common, while asthma and atopic dermatitis remain a significant public health concerns in adults in the context of rapid urbanization and shifts toward more Westernized eating patterns^2,3^. These trends have intensified growing interest in dietary exposures as potentially modifiable factors linked to allergic disease risk and persistence.

Dietary factors can influence allergic phenotypes through redox homeostasis, systemic and local inflammation, and immune regulation along interconnected mucosal pathways (often conceptualized as a gut-lung-skin axis)^4–6^. Accordingly, specific fatty acids, antioxidant vitamins, and selected minerals linked to antioxidant defenses and immune function may either reduce or worsen allergic inflammation^4,7^. Nevertheless, the epidemiologic literature shows inconsistent findings, and many studies have examined only a limited number of nutrients or focused on a single allergic outcome, thereby restricting comparisons across phenotypes^8^.

A nutrient-wide association study (NWAS) approach addresses these limitations by evaluating a broad panel of nutrients in parallel while controlling for multiple testing, typically via false discovery rate (FDR) procedures, similar to genome- or environment-wide studies^9,10^. Large, nationally representative health and nutrition surveys such as the Korea National Health and Nutrition Examination Survey (KNHANES) enable this framework by combining standardized 24-hour dietary recall data with information on physician-diagnosed allergic diseases and relevant covariates^11,12^. Using KNHANES 2016–2023 data, we conducted a cross-sectional analysis with survey-weighted logistic regression and FDR control to identify and compare nutrient associations with asthma, allergic rhinitis, and atopic dermatitis in Korean adults. This approach allowed us to prioritize nutrient signals for future prevention and management.

## Methods

### Study population

This study analyzed cross-sectional data from the KNHANES, a continuous nationwide survey representing the non-institutionalized Korean population through stratified, multistage probability sampling. The Korea Disease Control and Prevention Agency (KDCA) Review Board approves all KNHANES protocols, and all participants provide written informed consent.

Among 60,022 individuals who participated in KNHANES 2016–2023, we limited the dataset to adults aged ≥ 18 years who completed all three components (health interview, health examination, and nutrition survey) (*n* = 50,274). We then excluded participants based on the following criteria: pregnancy (*n* = 177), missing data for any nutrient intake variable (*n* = 8,364), missing responses to the allergic disease questionnaire (*n* = 2,413), implausible total energy intake (< 500 or > 5,000 kcal/day) (*n* = 608), and missing information on key covariates, including age, sex, body mass index (BMI), smoking status, household income, or education) (*n* = 904). The final analytic cohort comprised 37,808 adults.

For descriptive statistics, we created an “any allergic disease” indicator defined as having at least one of asthma, atopic dermatitis, or allergic rhinitis. For the primary regression analyses, we evaluated asthma, atopic dermatitis, and allergic rhinitis as separate outcomes. All analyses incorporated the complex survey design using KNHANES sampling weights, strata, and primary sampling units to generate nationally representative results.

### Assessment of allergic diseases

We ascertained allergic diseases using standardized KNHANES self-report questionnaires. Participants were classified as having asthma only when they reported a prior physician diagnosis (i.e., answered “yes” to the physician-diagnosis question). We used the same physician-diagnosis based classification for atopic dermatitis and allergic rhinitis. In regression models, we categorized individuals who answered “no” to all three physician-diagnosis items as having no allergic disease and used them as the reference group. Because KNHANES records only physician-diagnosed disease status, whether standardized diagnostic criteria were consistently applied by clinicians cannot be confirmed in this dataset.

### Dietary assessment and nutrient variables

Trained dietitians collected dietary intake using a 24-hour dietary recall interview, in which participants reported all foods and beverages consumed on the preceding day. The recall interview followed the standardized KNHANES dietary survey protocol implemented by the KDCA, and was conducted by trained interviewers using structured procedures^13^. We computed nutrient values using the Korean Food Composition Database maintained by the Rural Development Administration^14^.

For the nutrient-wide analysis, we prespecified 28 nutrients based on biological relevance and data availability within KNHANES. The nutrient set included macronutrients (carbohydrate, protein, total fat), fatty acid subtypes (saturated, monounsaturated, polyunsaturated, n-3, and n-6 fatty acids), cholesterol, total dietary fiber, minerals (calcium, phosphorus, sodium, potassium, magnesium, iron, and zinc), and vitamins (vitamin A as retinol activity equivalents, vitamin D, vitamin E, carotenoids, retinol, thiamin [vitamin B1], riboflavin [vitamin B2], niacin, folate, and vitamin C). We expressed nutrient intakes as absolute daily amounts (g/day for macronutrients, mg/day or µg/day for micronutrients).

### Statistical analysis

We incorporated KNHANES sampling weights, stratification variables, and clustering information in all analyses to generate nationally representative estimates. We summarized participant characteristics at baseline according to allergic disease status using standard descriptive comparisons. We evaluated associations between nutrient intakes and allergic outcomes (asthma, atopic dermatitis, and allergic rhinitis) using survey-weighted logistic regression models. For categorical variables, frequencies and weighted percentages accounting for the complex sampling design were calculated, and *p* values for differences in their distributions were obtained using Rao-Scott chi-square tests. For continuous variables, weighted means were estimated, and *p* values for differences in weighted means between groups were derived using survey-weighted linear regression models. These descriptive analyses were performed using SAS^®^ Analytics Pro version 9.4 or higher (SAS Institute Inc., Cary, NC, USA).

We treated each nutrient’s daily intake as the primary exposure and modeled it as a continuous predictor. We reported effect estimates as odds ratios (ORs) and 95% confidence intervals (CIs). Model 1 included age, sex, and BMI. Model 2, our primary model, further adjusted for smoking status, household income, and total daily energy intake (kcal/day). Total energy intake was included as a covariate in the regression model rather than adjusted using the residual method. To enhance comparability across nutrients, all nutrient intake variables were log-transformed to reduce skewness and improve model stability and subsequently standardized (z-scores) to a mean of 0 and standard deviation (SD) of 1 before modeling. The ORs therefore reflect the change in disease odds per 1-SD higher nutrient intake.

To control for multiple testing across 28 nutrients, we applied FDR correction within each allergic outcome using the Benjamini-Hochberg procedure. We defined statistical significance as an FDR-adjusted *q* value below 0.05. We also reported unadjusted (nominal) *p* values to aid interpretation of individual associations. Statistical analyses were performed using R software (version 4.3.2) with the survey package for complex-sample analyses.

## Results

A total of 60,022 individuals participated in KNHANES between 2016 and 2023. After excluding 9,748 participants younger than 18 years and 177 pregnant women, 50,274 adults were eligible for analysis.

**Fig. 1:**
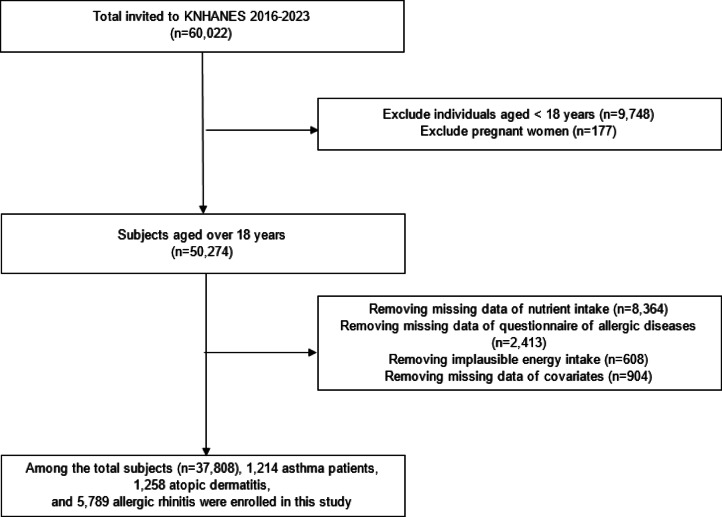
Study population flow diagram. Of 60,022 KNHANES participants with complete weighting information, we excluded individuals aged < 18 years, pregnant women, and participants with missing data on nutrient intake, allergic disease status, total energy intake, or key covariates, resulting in a final analytic sample of 37,808 adults.

We further excluded participants with missing nutrient intake information (*n* = 8,364), incomplete responses to the allergic disease questionnaire (*n* = 2,413), implausible total energy intake (< 500 or > 5,000 kcal/day; *n* = 608), and missing data on key covariates (age, sex, body mass index, smoking status, household income, and education level) (*n* = 904). The final analytic cohort consisted of 37,808 adults (Fig. 1). Of these, 7,314 participants reported at least one allergic disease. Specifically, 1,214 had asthma, 1,258 had atopic dermatitis, and 5,789 had allergic rhinitis, with overlap between conditions.

Overall, 19.3% of participants (*n* = 7,314) had at least one allergic disease, whereas 80.7% (*n* = 30,494) had none (Table 1). Participants with any allergic disease were younger on average than those without allergic disease (mean age, 41.9 vs. 49.5 years; *p* < 0.001). The proportion of women was higher in the allergic group than in the non-allergic group. (55.1% vs. 49.0%; *p* < 0.001). Mean BMI was slightly lower in participants with allergic disease than in those without (23.84 vs. 24.08 kg/m^2^; *p* < 0.001). Current smoking was less frequent in the allergic group than in the non-allergic group (16.7% vs. 19.3%; *p* < 0.001). Household income distribution showed no significant difference between groups (*p* = 0.442).

**Table 1:** Baseline characteristics of study participants according to allergic diseases status.

Variable	Category	Non-allergic diseases (*n* = 30,494)	Any allergic diseases (*n* = 7,314)	Asthma (*n* = 1,214)	Atopic dermatitis (*n* = 1,258)	Allergic rhinitis (*n* = 5,789)	*P* value (allergic vs. non-allergic)
Age, years		49.46	41.93	48.95	33.36	41.70	< 0.001
Age group, n (%)	18–29	2,866 (14.53)	1,512 (28.40)	172 (23.25)	593 (56.06)	1,106 (25.99)	
	30–39	3,780 (15.50)	1,432 (21.75)	146 (16.40)	247 (20.22)	1,235 (23.03)	
	40–49	5,284 (19.85)	1,387 (18.54)	128 (11.63)	125 (8.50)	1,245 (20.74)	
	50–59	5,894 (21.24)	1,173 (15.46)	156 (12.95)	114 (7.66)	1,020 (16.88)	
	60–69	6,506 (15.32)	963 (8.53)	238 (14.39)	92 (3.68)	732 (8.31)	
	70–79	4,637 (10.35)	655 (5.73)	266 (15.23)	68 (2.97)	376 (4.26)	
	≥ 80	1,527 (3.21)	192 (1.58)	108 (6.16)	19 (0.91)	75 (0.78)	
Sex, n (%)	Male / Female	13,425 / 17,069 (50.99 / 49.01)	2,721 / 4,593 (44.91 / 55.09)	475 / 739 (47.50 / 52.50)	538 / 720 (49.11 / 50.89)	2,059 / 3,730 (43.32 / 56.68)	< 0.001
BMI, kg/m²		24.08	23.84	24.41	23.84	23.76	< 0.001
Current smoking, n (%)	Yes	4,949 (19.29)	1,015 (16.64)	204 (20.56)	225 (19.87)	745 (15.50)	< 0.001
Household income, n (%)							0.442
	1 (lowest)	7,429 (23.85)	1,827 (24.51)	348 (28.22)	347 (26.99)	1,387 (23.58)	
	2 (low–middle)	7,692 (24.87)	1,777 (24.15)	299 (23.22)	301 (23.12)	1,408 (24.42)	
	3 (middle–high)	7,722 (25.49)	1,821 (24.89)	281 (23.45)	283 (22.49)	1,461 (25.23)	
	4 (highest)	7,651 (25.80)	1,889 (26.45)	286 (25.11)	327 (27.40)	1,533 (26.76)	
Hypertension, n (%)	Yes	8,537 (22.35)	1,343 (13.95)	422 (26.67)	142 (7.28)	909 (12.63)	< 0.001
Diabetes mellitus, n (%)	Yes	3,560 (9.42)	474 (4.82)	169 (10.37)	45 (2.31)	310 (4.22)	< 0.001
Total cholesterol, mg/dL		191.30	190.80	187.33	187.36	191.78	0.384
HDL cholesterol, mg/dL		52.65	54.74	52.72	55.09	55.07	< 0.001
Triglycerides, mg/dL		134.34	121.81	125.12	118.92	120.53	< 0.001

Values are presented as weighted means or frequencies (weighted percentages). All analyses accounted for the complex survey design using sampling weights, stratification, and clustering. Weighted percentages are calculated within each column. *P* values compare participants with any allergic disease to those without allergic disease and were obtained using survey-weighted linear regression for continuous variables and the Rao-Scott chi-square test for categorical variables.

In contrast, cardio-metabolic comorbidities showed distinct patterns: hypertension was less commonly in participants with any allergic disease than in those without (14.0% vs. 22.4%; *p* < 0.001), with the highest prevalence observed among individuals with asthma (20.6%). Diabetes mellitus showed a similar pattern, with a higher prevalence in asthma than in atopic dermatitis or allergic rhinitis (*p* < 0.001).

Total cholesterol levels did not differ significantly between the allergic and non-allergic groups (190.80 vs. 191.30 mg/dL, *p* = 0.384). However, participants with allergic disease showed a more favorable lipid profile, with higher high-density lipoprotein (HDL) cholesterol levels (54.74 vs. 52.65 mg/dL) and lower triglycerides (TG) concentrations (121.81 vs. 134.34 mg/dL). Both differences were statistically significance (*p* < 0.001).

As shown in Table 2, individuals with any allergic disease consumed marginally more total energy than those without allergic disease (1,941.66 vs. 1,910.86 kcal/day, *p* = 0.011). The allergic group also reported higher intakes of protein and total fat (protein 73.53 vs. 70.90 g/day; fat 52.17 vs. 46.82 g/day; both *p* < 0.001). Multiple lipid-related measures, including saturated fat (SFA), monounsaturated fatty acids (MUFA), polyunsaturated fatty acids (PUFA), and n-6 fatty acids (N6), showed consistent differences. Cholesterol and total sugar intakes were also higher in participants with allergic disease (both *p* < 0.001), whereas total carbohydrate (CHO) and total dietary fiber (TDF) intakes were lower (both *p* < 0.001).

**Table 2:** Dietary energy and nutrient intakes according to allergic disease status.

Nutrient	Total (*n* = 37,808)	Non-allergic diseases (*n* = 30,494)	Any allergic diseases (*n* = 7,314)	Asthma (*n* = 1,214)	Atopic dermatitis (*n* = 1,258)	Allergic rhinitis (*n* = 5,789)	*P* value (allergic vs. non-allergic)
Total energy (kcal/day)	1,917.36	1,910.86	1,941.66	1,854.06	1,979.71	1,943.23	0.011
Protein (g/day)	71.46	70.90	73.53	68.58	75.67	73.86	< 0.001
Total fat (g/day)	47.95	46.82	52.17	46.07	56.02	52.55	< 0.001
Saturated fat (g/day)	15.30	14.87	16.88	14.73	18.32	17.00	< 0.001
MUFA (g/day)	15.50	15.10	17.00	14.87	18.30	17.13	< 0.001
PUFA (g/day)	12.29	12.09	13.05	11.88	13.89	13.13	< 0.001
n–3 fatty acids (g/day)	1.88	1.88	1.87	1.79	1.84	1.89	0.950
n–6 fatty acids (g/day)	10.38	10.18	11.14	10.05	12.00	11.20	
Cholesterol (mg/day)	254.07	250.01	269.27	246.11	275.41	272.02	
Carbohydrate (g/day)	277.20	278.47	272.44	274.31	267.70	272.00	
Dietary fiber (g/day)	25.11	25.43	23.90	23.69	21.99	24.10	
Sugars (g/day)	59.76	59.16	61.99	59.61	62.38	62.33	
Calcium (mg/day)	505.45	505.44	505.49	484.91	487.96	510.98	0.990
Phosphorus (mg/day)	1,062.30	1,061.20	1,066.43	1,015.62	1,055.08	1,073.30	0.439
Sodium (mg/day)	3,327.10	3,330.01	3,316.23	3,175.02	3,251.99	3,342.88	0.637
Potassium (mg/day)	2,756.82	2,770.43	2,705.88	2,580.51	2,549.71	2,740.81	0.001
Magnesium (mg/day)	308.53	310.85	299.84	289.77	283.13	302.25	< 0.001
Iron (mg/day)	10.57	10.58	10.52	10.64	10.18	10.55	0.538
Zinc (mg/day)	10.27	10.27	10.28	9.81	10.00	10.34	0.969
Vitamin A (µg RAE/day)	389.49	387.89	395.48	395.23	373.86	397.29	0.189
Vitamin D (µg/day)	3.13	3.14	3.11	3.00	3.09	3.11	0.699
Vitamin E (mg α-TE/day)	6.86	6.81	7.07	6.59	7.12	7.11	< 0.001
β-carotene (µg/day)	2,813.34	2,841.60	2,707.60	2,693.20	2,398.77	2,746.91	0.002
Retinol (µg/day)	154.08	150.29	168.26	169.77	172.77	166.75	< 0.001
Vitamin B1 (mg/day)	1.22	1.22	1.22	1.18	1.21	1.23	0.533
Vitamin B2 (mg/day)	1.60	1.58	1.66	1.55	1.65	1.68	< 0.001
Niacin (mg/day)	12.91	12.80	13.29	12.50	13.42	13.37	< 0.001
Folate (µg DFE/day)	316.61	320.04	303.78	301.14	278.60	306.71	< 0.001
Vitamin C (mg/day)	65.77	65.83	65.56	59.87	57.91	67.06	0.858

Values are presented as weighted mean daily intakes according to allergic disease status. *P* values compare participants with any allergic disease and those without allergic disease and were obtained using survey-weighted linear regression models accounting for the complex sampling design. All nutrient intakes are unadjusted values derived from the 24-hour dietary recall.

For minerals, the allergic group showed slightly lower intakes of potassium (K) and magnesium (MG) (K, 2,705.88 vs. 2,770.43 mg/day, *p* = 0.001; Mg, 299.84 vs. 310.85 mg/day, *p* < 0.001). In contrast, calcium, phosphorus, sodium, iron, and zinc showed no significant differences between groups (all *p* > 0.05).

Vitamin intake patterns varied. Participants with allergic disease consumed more vitamin E (VITE), beta-carotene, retinol, riboflavin (vitamin B2, B2), and niacin (NIAC) (all *p* < 0.05), whereas folate intake was lower (303.78 vs. 320.04 µg DFE/day; *p* < 0.001). Intakes of vitamin A (VA_RAE, as retinol activity equivalents), vitamin D, thiamin (vitamin B1), and vitamin C showed no meaningful differences between groups.

**Fig. 2:**
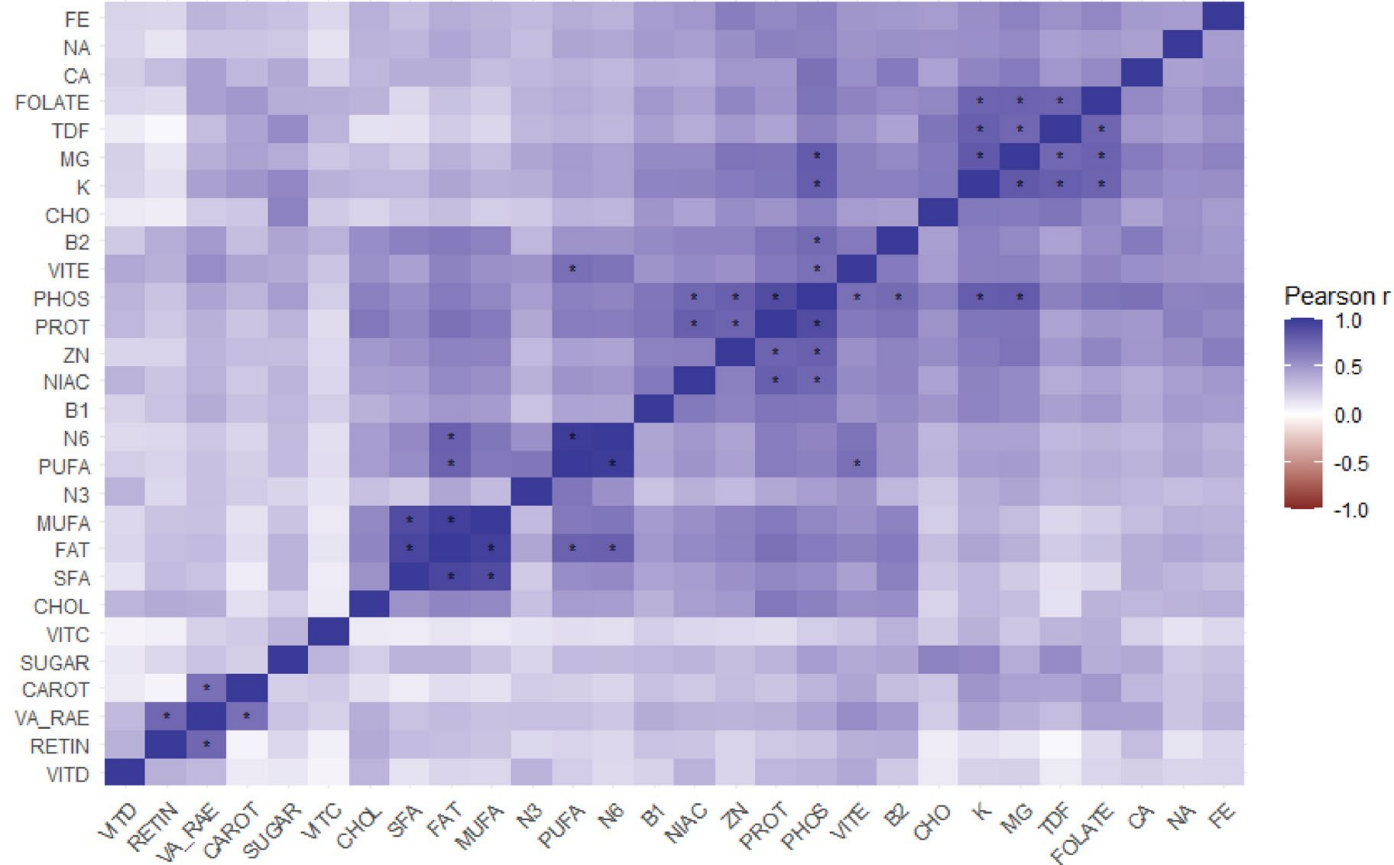
Pearson correlation heatmap of energy-adjusted nutrient intakes. Pearson correlation heatmap of energy-adjusted intakes of 28 nutrients. Blue indicates positive correlations and red negative correlations; darker color reflect stronger associations. Strong correlations (|r| $\:\ge\:$ 0.70) are marked as asterisk(*). Nutrients are grouped to highlight clusters reflecting shared dietary sources. FE, iron; Na, sodium; CA, calcium; FOLATE, folate; TDF, total dietary fiber; MG, magnesium; K, potassium; CHO, carbohydrate; B2, riboflavin; VITE, vitamin E; PHOS, phosphorus; PROT, protein; ZN, zinc; NIAC, niacin; B1, thiamin; N6, n-6 fatty acids; PUFA, polyunsaturated fatty acids; N3, n-3 fatty acids; MUFA, monounsaturated fatty acids; FAT, total fat; SFA, saturated fatty acids; CHOL, cholesterol; VITC, vitamin C; SUGAR, total sugars; CAROT, carotenoids; VA_RAE, vitamin A (retinol activity equivalents); RETIN, retinol; VITD, vitamin D.

Figure 2 shows the correlation structure among energy-adjusted nutrients. Fat-related variables (total fat [FAT], SFA, MUFA, PUFA, n-3, and n-6) showed strong positive correlations (|r| ≥ 0.70). Another cluster comprised vitamin A, retinol, and carotenoids. Minerals such as calcium, magnesium, and phosphorus showed moderate correlations, whereas correlations across biochemical classes were generally weaker (|r| < 0.70). These patterns suggest that some nutrient-disease associations may reflect broader dietary patterns rather than isolated effects of single nutrients.

**Fig. 3:**
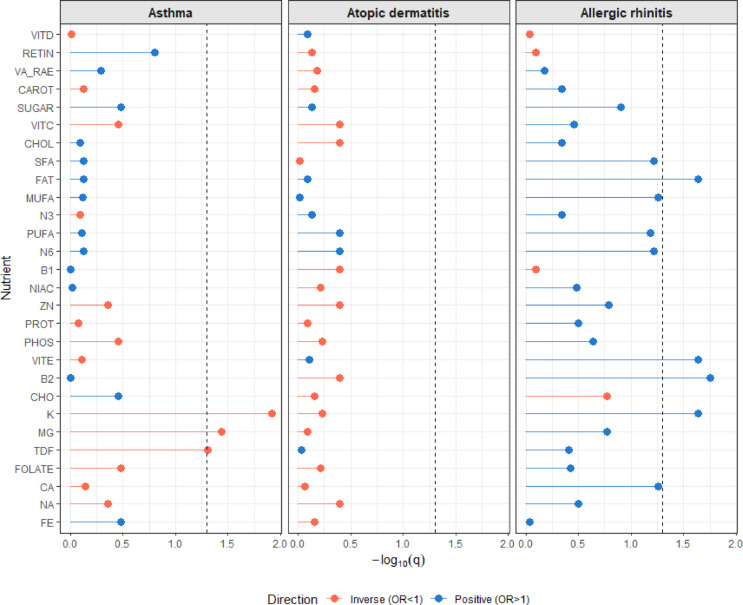
Nutrient-wide associations with allergic diseases (-log10(*q*) plot). Nutrient-wide associations of energy-adjusted nutrient intakes with allergic rhinitis, asthma, and atopic dermatitis. Each point represents the association between a 1-standard-deviation (SD) increase in intake of a given nutrient and the odds of each outcome from multivariable logistic regression models (Model 2). The x-axis shows –log10(*q*), where q is the false discovery rate (FDR)-adjusted *p* value; larger values indicate stronger statistical evidence. The vertical dashed line denotes the FDR threshold of *q* = 0.05. Points are colored by direction of association; red for inverse associations (odds ratio < 1) and blue for positive for positive associations (odds ratio > 1). Nutrients are displayed on the y-axis in the same order across panels for comparison.

In the nutrient-wide analyses, we examined 28 energy-adjusted nutrients for each allergic outcome using multivariable survey-weighted logistic regression (Models 1 and 2) (Fig. 3). In the fully adjusted model, we identified three FDR-robust associations for asthma (*q* ≤ 0.05): TDF, K, and MG (Table 3). All three showed inverse associations with asthma, with ORs per 1-SD higher intake ranging from 0.8 to 0.9, indicating lower asthma odds at greater intakes.

**Table 3:** Nutrient-wide associations between energy-adjusted nutrient intakes and asthma.

Nutrient	OR (Model 1)	95% CI	*p* value	*q* value	OR (Model 2)	95% CI	*p* value	*q* value
FAT	0.965	0.89–1.04	0.360	0.577	1.036	0.94–1.14	0.481	0.748
PROT	0.935	0.86–1.02	0.140	0.327	0.976	0.86–1.11	0.713	0.832
SFA	0.977	0.90–1.05	0.546	0.695	1.037	0.95–1.13	0.434	0.748
MUFA	0.970	0.90–1.04	0.417	0.615	1.029	0.94–1.12	0.517	0.761
PUFA	0.971	0.90–1.05	0.448	0.628	1.026	0.94–1.12	0.579	0.772
N3	0.952	0.88–1.03	0.240	0.471	0.982	0.90–1.07	0.663	0.807
N6	0.976	0.91–1.05	0.529	0.695	1.033	0.95–1.13	0.456	0.748
CHOL	0.981	0.90–1.07	0.653	0.703	1.022	0.93–1.12	0.638	0.807
CHO	0.983	0.92–1.06	0.642	0.703	1.095	0.98–1.23	0.124	0.346
TDF	0.871	0.80–0.94	0.001	0.008	0.873	0.79–0.96	0.005	**0.050**
SUGAR	1.007	0.95–1.07	0.806	0.806	1.064	0.99–1.14	0.082	0.327
CA	0.933	0.86–1.01	0.087	0.244	0.960	0.88–1.05	0.358	0.715
PHOS	0.907	0.83–0.99	0.021	0.120	0.903	0.80–1.02	0.108	0.346
NA	0.923	0.85–1.00	0.056	0.197	0.943	0.86–1.03	0.187	0.437
K	0.852	0.78–0.93	0.000	0.005	0.818	0.73–0.91	0.000	**0.012**
MG	0.854	0.78–0.94	0.001	0.008	0.825	0.73–0.93	0.003	**0.036**
FE	1.021	0.94–1.10	0.604	0.703	1.075	1.00–1.16	0.058	0.327
ZN	0.913	0.84–0.99	0.034	0.160	0.927	0.83–1.03	0.177	0.437
VA_RAE	1.017	0.95–1.09	0.636	0.703	1.040	0.97–1.11	0.237	0.510
VITD	0.978	0.88–1.09	0.678	0.703	0.994	0.90–1.10	0.897	0.966
VITE	0.940	0.87–1.02	0.116	0.296	0.976	0.90–1.06	0.570	0.772
CAROT	0.948	0.87–1.03	0.214	0.461	0.971	0.89–1.05	0.478	0.748
RETIN	1.046	0.99–1.10	0.080	0.244	1.058	1.01–1.11	0.023	0.158
B1	0.961	0.88–1.04	0.347	0.577	1.002	0.92–1.10	0.959	0.995
B2	0.955	0.88–1.03	0.252	0.471	1.000	0.91–1.10	0.995	0.995
NIAC	0.963	0.89–1.05	0.371	0.577	1.009	0.92–1.11	0.850	0.951
FOLATE	0.892	0.81–0.98	0.015	0.107	0.904	0.81–1.01	0.077	0.327
VITC	0.914	0.83–1.00	0.051	0.197	0.935	0.86–1.02	0.112	0.346

Odds ratio (ORs) and 95% confidence intervals (CIs) for asthma per 1-standard-deviation (SD) increase in energy-adjusted intake of each nutrient, estimated using logistic regression. Model 1 was adjusted for age, sex, and body mass index (BMI). Model 2 was additionally adjusted for total energy intake, smoking status, and household income. *P* values and *q* values (false discovery rate (FDR)-adjusted *p* values) are shown for each model. Nutrients with *q* < 0.05 in Model 2 are highlighted in bold.

FAT, total fat; PROT, protein; SFA, saturated fatty acids; MUFA, monounsaturated fatty acids; PUFA, polyunsaturated fatty acids; N3, n-3 fatty acids; N6, n-6 fatty acids; CHOL, cholesterol; CHO, carbohydrate; TDF, total dietary fiber; SUGAR, total sugars; CA, calcium; PHOS, phosphorus; NA, sodium; K, potassium; MG, magnesium; FE, iron; ZN, zinc; VA_RAE, vitamin A (retinol activity equivalents); VITD, vitamin D; VITE, vitamin E; CAROT, carotenoids; RETIN, retinol; B1, thiamin; B2, riboflavin; NIAC, niacin; FOLATE, folate; VITC, vitamin C.

For atopic dermatitis, no nutrient met the FDR criterion in Model 2 (Table 4). For allergic rhinitis, four nutrients reached FDR significance (*q* < 0.05) ─FAT, K, VITE, and B2─ and all showed positive associations with disease odds (Table 5). Several additional fat-related nutrients (including SFA, MUFA, PUFA, and N6) showed similar positive directions, with *q* values marginally above 0.05. These findings were directionally consistent with positive associations.

**Table 4:** Nutrient-wide associations between energy-adjusted nutrient intakes and allergic rhinitis.

Nutrient	OR (Model 1)	95% CI	*p* value	*q* value	OR (Model 2)	95% CI	*p* value	*q* value
FAT	1.068	1.03–1.10	< 0.001	< 0.001	1.079	1.03–1.13	0.003	**0.023**
PROT	1.051	1.01–1.09	0.006	0.012	1.042	0.98–1.10	0.170	0.317
SFA	1.057	1.02–1.09	< 0.001	0.002	1.054	1.01–1.10	0.017	0.061
MUFA	1.061	1.03–1.10	< 0.001	0.001	1.06	1.01–1.11	0.012	0.055
PUFA	1.057	1.03–1.09	< 0.001	0.001	1.045	1.01–1.09	0.021	0.065
N3	1.033	1.00–1.06	0.035	0.048	1.016	0.98–1.05	0.362	0.456
N6	1.057	1.03–1.09	< 0.001	0.001	1.046	1.01–1.09	0.017	0.061
CHOL	1.036	1.00–1.07	0.025	0.037	1.018	0.98–1.06	0.345	0.456
CHO	1.011	0.97–1.05	0.564	0.608	0.951	0.90–1.01	0.078	0.169
TDF	1.047	1.01–1.09	0.015	0.028	1.024	0.98–1.07	0.280	0.391
SUGAR	1.055	1.02–1.09	0.002	0.005	1.042	1.00–1.08	0.045	0.125
CA	1.059	1.03–1.09	< 0.001	0.001	1.046	1.01–1.08	0.010	0.055
PHOS	1.055	1.02–1.09	0.002	0.005	1.047	0.99–1.11	0.115	0.231
NA	1.041	1.01–1.08	0.021	0.033	1.027	0.99–1.07	0.183	0.320
K	1.075	1.04–1.11	< 0.001	< 0.001	1.076	1.03–1.13	0.003	**0.023**
MG	1.057	1.02–1.09	0.002	0.005	1.043	1.00–1.09	0.076	0.169
FE	1.025	0.99–1.06	0.116	0.147	1.003	0.97–1.04	0.888	0.921
ZN	1.053	1.02–1.09	0.002	0.005	1.045	1.00–1.09	0.064	0.163
VA_RAE	1.022	0.99–1.05	0.136	0.166	1.009	0.98–1.04	0.569	0.664
VITD	1.008	0.98–1.04	0.618	0.640	0.998	0.97–1.03	0.921	0.921
VITE	1.066	1.03–1.10	< 0.001	< 0.001	1.061	1.02–1.10	0.003	**0.023**
CAROT	1.027	0.99–1.07	0.165	0.192	1.016	0.98–1.05	0.374	0.456
RETIN	1.005	0.98–1.03	0.743	0.743	0.995	0.97–1.03	0.737	0.806
B1	1.020	0.99–1.05	0.238	0.267	0.994	0.96–1.03	0.749	0.806
B2	1.073	1.04–1.11	< 0.001	< 0.001	1.077	1.03–1.12	< 0.001	**0.018**
NIAC	1.041	1.01–1.08	0.017	0.028	1.027	0.99–1.07	0.199	0.328
FOLATE	1.046	1.01–1.08	0.017	0.028	1.025	0.98–1.07	0.256	0.377
VITC	1.027	1.00–1.06	0.079	0.105	1.019	0.99–1.05	0.226	0.352

Odds ratios (ORs) and 95% confidence intervals (CIs) for allergic rhinitis per 1-standard-deviation (SD) increase in log-transformed, energy-adjusted intake of each nutrient, estimated using logistic regression. Model 1 was adjusted for age, sex and body mass index (BMI). Model 2 was additionally adjusted for total energy intake, smoking status, and household income. *q* values are false discovery rate (FDR)-adjusted *p* values calculated across all 28 nutrients for Model 2; nutrients with *q* < 0.05 in Model 2 are highlighted in bold.

FAT, total fat; PROT, protein; SFA, saturated fatty acids; MUFA, monounsaturated fatty acids; PUFA, polyunsaturated fatty acids; N3, n-3 fatty acids; N6, n-6 fatty acids; CHOL, cholesterol; CHO, carbohydrate; TDF, total dietary fiber; SUGAR, total sugars; CA, calcium; PHOS, phosphorus; NA, sodium; K, potassium; MG, magnesium; FE, iron; ZN, zinc; VA_RAE, vitamin A (retinol activity equivalents); VITD, vitamin D; VITE, vitamin E; CAROT, carotenoids; RETIN, retinol; B1, thiamin; B2, riboflavin; NIAC, niacin; FOLATE, folate; VITC, vitamin C.

**Table 5:** Nutrient-wide associations between energy-adjusted nutrient intakes and atopic dermatitis.

Nutrient	OR (Model 1)	95% CI	*p* value	*q* value	OR (Model 2)	95% CI	*p* value	*q* value
FAT	0.997	0.94–1.06	0.914	0.914	1.020	0.93–1.12	0.684	0.813
PROT	0.978	0.92–1.04	0.480	0.746	0.977	0.89–1.08	0.645	0.813
SFA	0.988	0.93–1.05	0.692	0.837	0.997	0.92–1.08	0.946	0.962
MUFA	0.990	0.93–1.05	0.748	0.837	1.002	0.92–1.09	0.962	0.962
PUFA	1.027	0.97–1.09	0.358	0.626	1.059	0.99–1.14	0.115	0.404
N3	1.014	0.95–1.08	0.688	0.837	1.024	0.96–1.09	0.471	0.733
N6	1.027	0.97–1.08	0.339	0.626	1.059	0.99–1.14	0.109	0.404
CHOL	0.940	0.88–1.00	0.053	0.497	0.932	0.87–1.00	0.050	0.404
CHO	0.969	0.91–1.03	0.329	0.626	0.959	0.87–1.06	0.396	0.693
TDF	0.994	0.93–1.07	0.874	0.907	1.007	0.93–1.10	0.869	0.936
SUGAR	1.011	0.95–1.08	0.732	0.837	1.026	0.96–1.10	0.467	0.733
CA	0.983	0.92–1.06	0.644	0.837	0.988	0.91–1.07	0.762	0.853
PHOS	0.962	0.90–1.03	0.236	0.626	0.933	0.84–1.04	0.201	0.590
NA	0.944	0.88–1.01	0.113	0.528	0.930	0.85–1.01	0.095	0.404
K	0.957	0.89–1.03	0.213	0.626	0.941	0.85–1.04	0.211	0.590
MG	0.978	0.92–1.04	0.515	0.759	0.982	0.90–1.08	0.697	0.813
FE	0.961	0.89–1.03	0.286	0.626	0.960	0.88–1.05	0.375	0.693
ZN	0.945	0.88–1.01	0.096	0.528	0.916	0.83–1.01	0.078	0.404
VA_RAE	0.954	0.88–1.04	0.277	0.626	0.955	0.87–1.04	0.304	0.656
VITD	1.014	0.95–1.09	0.698	0.837	1.017	0.95–1.09	0.628	0.813
VITE	1.006	0.94–1.07	0.871	0.907	1.025	0.94–1.12	0.562	0.787
CAROT	0.968	0.91–1.03	0.323	0.626	0.970	0.91–1.04	0.363	0.693
RETIN	0.965	0.88–1.06	0.467	0.746	0.967	0.88–1.07	0.502	0.739
B1	0.922	0.86–0.99	0.025	0.474	0.897	0.82–0.98	0.015	0.404
B2	0.940	0.88–1.01	0.072	0.501	0.922	0.85–1.00	0.060	0.404
NIAC	0.961	0.90–1.02	0.216	0.626	0.954	0.88–1.03	0.250	0.614
FOLATE	0.956	0.89–1.03	0.239	0.626	0.952	0.87–1.04	0.263	0.614
VITC	0.934	0.88–0.99	0.034	0.474	0.933	0.87–1.00	0.037	0.404

Odds ratios (ORs) and 95% confidence intervals (CIs) for atopic dermatitis per 1-standard-deviation (SD) increase in log-transformed, energy-adjusted intake of each nutrient, estimated using logistic regression. Model 1 was adjusted for age, sex and body mass index (BMI). Model 2 was additionally adjusted for total energy intake, smoking status, and household income. *q* values are false discovery rate (FDR)-adjusted *p* values calculated across all 28 nutrients for Model 2; nutrients with *q* < 0.05 in Model 2 are highlighted in bold.

FAT, total fat; PROT, protein; SFA, saturated fatty acids; MUFA, monounsaturated fatty acids; PUFA, polyunsaturated fatty acids; N3, n-3 fatty acids; N6, n-6 fatty acids; CHOL, cholesterol; CHO, carbohydrate; TDF, total dietary fiber; SUGAR, total sugars; CA, calcium; PHOS, phosphorus; NA, sodium; K, potassium; MG, magnesium; FE, iron; ZN, zinc; VA_RAE, vitamin A (retinol activity equivalents); VITD, vitamin D; VITE, vitamin E; CAROT, carotenoids; RETIN, retinol; B1, thiamin; B2, riboflavin; NIAC, niacin; FOLATE, folate; VITC, vitamin C.

**Fig. 4:**
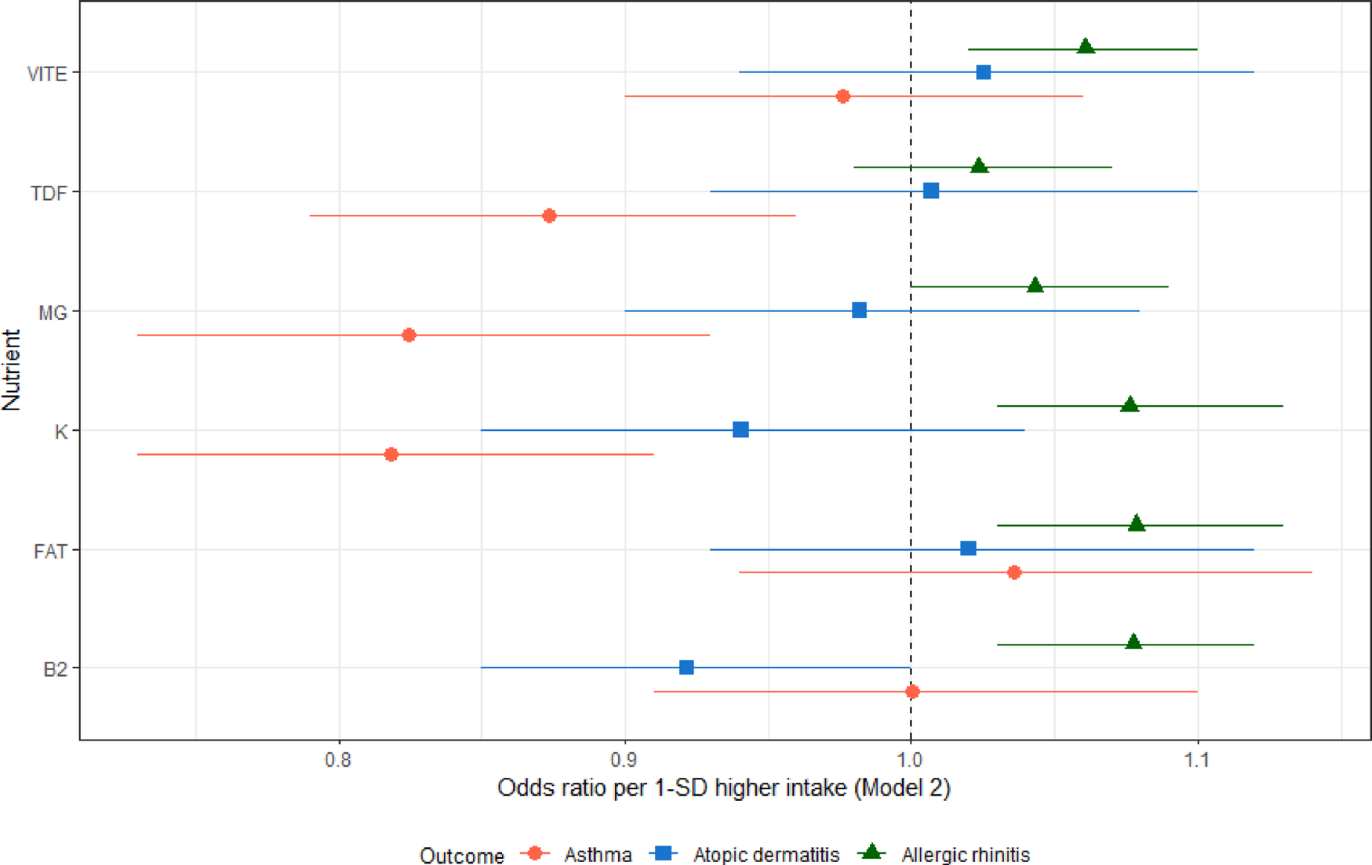
Forest plot of fully adjusted odds ratios (ORs) for asthma, atopic dermatitis, and allergic rhinitis per 1-standard-deviation (SD) increase in nutrient intake. Nutrients shown are those that passed the false discovery rate (FDR) threshold (*q* < 0.05) in at least one outcome. Points indicate odds ratios from Model 2 (adjusted for age, sex, body mass index (BMI), total energy intake, smoking status, and household income); horizontal lines show 95% confidence intervals. A vertical dashed line marks the null value (OR = 1.0). Estimates for asthma (red circles), atopic dermatitis (blue squares), and allergic rhinitis (green triangles) are displayed on the same row for each nutrient.

Figure 4 summarizes the Model 2 estimates for the six FDR-significant nutrients (FAT, K, MG, TDF, VITE and B2), showing ORs per 1-SD higher intake. For asthma, higher TDF, K, and MG intakes consistently showed reduced odds (OR < 1.0), with CIs excluding the null. In contrast, greater intakes of FAT, K, VITE, and B2 showed higher odds of allergic rhinitis, with effect sizes modestly above 1.0. For atopic dermatitis, estimates for these nutrients clustered around the null, with no associations reaching significance after full adjustment.

## Discussion

Although the population burden of allergic diseases in Korea continues to increase, prior research on nutrient-disease relationships has largely focused on isolated nutrients and shows heterogeneous findings across allergic outcomes^8^. To address these limitations, we performed a nutrient-wide association analysis using KNHANES 2016–2023. We evaluated 28 daily nutrient intakes in relation to physician-diagnosed asthma, atopic dermatitis, and allergic rhinitis in a nationally representative sample of Korean adults. In fully adjusted, survey-weighted models with FDR control, higher intakes of TDF, K, and MG showed reduced odds of asthma, while higher intakes of FAT, VITE, and K showed increased odds of allergic rhinitis. No nutrients reached the FDR significance threshold for atopic dermatitis. This null finding may reflect weaker diet-related signals, limited statistical power, or greater etiologic heterogeneity for this phenotype. Figures 3 and 4 show that the direction and strength of associations differed across the three allergic conditions, suggesting distinct nutrient-related patterns for asthma versus allergic rhinitis.

Asthma showed the clearest inverse signals, particularly for TDF, K, and MG. Prior work has linked higher fiber intake to improved asthma status. For example, the NutriNet-Santé e-cohort showed that greater total fiber consumption was associated with lower asthma symptom scores and better asthma control in adults^15^. Epidemiologic studies and clinical trials also support a potential role for magnesium. Lower dietary magnesium intake is associated with poorer lung function and heightened bronchial hyper-responsiveness. Some studies report improved respiratory outcomes following higher magnesium intake or supplementation^16–18^. Previous observations suggest that adults with asthma, especially those receiving oral beta-2 agonists, may show lower potassium and altered magnesium status compared with healthy controls. A recent retrospective cohort study also reported that higher dietary potassium intake was associated with lower all-cause mortality among adults with asthma^19,20^.

Several possible pathways could explain these findings. Mechanistic and experimental evidence shows that greater consumption of fermentable fiber can increase microbial generation of short-chain fatty acids (SCFAs), including acetate, propionate, and butyrate. SCFAs may influence systemic immunity by shaping bone marrow hematopoiesis and downstream airway immune phenotypes. This process can attenuate allergic airway inflammation through gut-lung crosstalk^21–23^. In addition, magnesium and potassium help maintain transmembrane electrical gradients and regulate airway smooth muscle contractility. In asthma, low magnesium status is linked to heightened bronchial hyper-responsiveness, wheezing, reduced lung function, and more frequent or severe exacerbations. Low potassium levels may also contribute to exacerbation risk^16,20,24,25^. These biological mechanisms may be relevant to the inverse associations observed between dietary fiber, magnesium, and potassium intake and prevalent asthma in this study.

Allergic rhinitis showed a clearly distinct nutrient-wide profile compared with asthma. After FDR adjustment, higher intakes of FAT, VITE, B2, and K showed higher odds of allergic rhinitis. This pattern is broadly consistent with previous studies reporting associations between high-fat or Western-style dietary patterns and allergic rhinitis^26,27^. In contrast, we did not anticipate the positive signals for vitamin E and riboflavin, as both nutrients have antioxidant and immunomodulatory functions and are commonly viewed as potentially beneficial^28–30^.

Indeed, experimental and some clinical data suggest that vitamin E may reduce airway inflammation and improve symptoms in certain allergic settings^31–33^. Nevertheless, human evidence for rhinitis remains inconsistent. Several observational studies and intervention trials, including one in perennial allergic rhinitis, found no consistent advantage of higher dietary vitamin E intake or supplementation for rhinitis-related outcomes^34–36^.

The literature on riboflavin is similarly heterogeneous. For example, one pediatric study reported lower allergy prevalence among children with higher vitamin B2 intake. In contrast, a study of Korean infants found that intakes exceeding recommended levels for multiple nutrients, including riboflavin, showed higher odds of allergic rhinitis^37,38^.

The positive associations of allergic rhinitis with vitamin E and riboflavin intake in Korean adults may be better interpreted as markers of correlated food sources, broader dietary patterns, or accompanying health behaviors. These findings do not necessarily indicate a direct adverse effect of these vitamins.

Potassium showed a positive association with allergic rhinitis even though it showed an inverse association with asthma within the same analytic sample. One plausible explanation is that higher potassium intake may serve as a marker of broader eating patterns in this population, such as greater consumption of fruits, vegetables, and composite mixed dishes. These patterns may correlate with lifestyle factors or environmental exposures relevant to allergic rhinitis risk^39,40^. This heterogeneity suggests that upper- and lower-airway allergic conditions may not share identical nutritional correlates. It also indicates that single-nutrient signals may partly reflect underlying dietary patterns or behavioral changes related to disease presence. Residual confounding and reverse causation should also be considered, as the cross-sectional design does not allow causal interpretation of the potassium findings.

In contrast to asthma and allergic rhinitis, no nutrient met the FDR significance threshold for atopic dermatitis among adults, although several nutrients showed modest inverse associations at nominal significance levels. This overall null pattern is biologically plausible. Atopic dermatitis is strongly influenced by genetic determinants of skin-barrier integrity, early-life environmental and microbial influences, and immune dysregulation. These factors may diminish the detectable contribution of single nutrient intakes measured in adulthood^41,42^.

However, some previous studies conducted in Asian populations have reported associations between dietary factors and atopic dermatitis, including studies suggesting links between dietary patterns, micronutrient intake, and allergic outcomes^43,44^. Recent studies has also suggested that food-group exposures, such as frequent fast food intake, and aspects of protein quality may be relevant to atopic dermatitis risk in Asian populations^45,46^. Differences between these studies and the present analysis may reflect methodological variation, including differences in dietary assessment methods, outcome definitions, population characteristics, and study design. Moreover, prior studies have typically examined habitual intake or food group-based exposures. In contrast, we used single-day nutrient level estimates, which may partly account for differences in effect sizes and associations. Atopic dermatitis in KNHANES is identified through self-reported lifetime physician diagnosis and is less common than asthma or allergic rhinitis. These factors likely limited statistical power and increased vulnerability to recall error and outcome misclassification. This may explain why no nutrient reached the FDR significance threshold for atopic dermatitis in the present analysis. Further prospective studies incorporating detailed phenotyping, repeated dietary measurements, and larger numbers of adults with atopic dermatitis are needed to clarify whether specific nutrients play an etiologic or disease-modifying role in adult atopic dermatitis. Taken together, these findings should be interpreted as cross-sectional associations rather than evidence of causal nutrient effects, and residual confounding and reverse causation cannot be excluded.

This study has several strengths. It uses a large, nationally representative sample of Korean adults from KNHANES 2016–2023, supporting population-level generalizability. Second, we analyzed asthma, atopic dermatitis, and allergic rhinitis within a single, consistent framework, enabling direct comparisons across outcomes. In addition, we applied a nutrient-wide association strategy encompassing 28 nutrients and explicitly addressed multiple comparisons using FDR control.

Several limitations should be considered. The cross-sectional design precludes causal inference and remains vulnerable to reverse causation. We assessed dietary exposure using a single 24-hour recall, which captures only one day of intake and is susceptible to day-to-day variability as well as weekday-weekend and seasonal variation. Such measurement error may attenuate true diet-disease associations. Although a single 24-hour recall does not reflect usual intake at the individual level, it remains useful for comparing average intake patterns across population groups in a large nationally representative surveys. Questionnaire-based definitions of allergic diseases have been used in Korean population-based studies, supporting their applicability in epidemiologic research^47^. Moreover, because allergic diseases often develop earlier in life, dietary intake measured in adulthood may not reflect exposures relevant to disease onset. Accordingly, the present findings should be interpreted as associations with current disease status rather than determinants of disease development. In addition, allergic outcomes were based on only self-reported physician diagnosis, without objective sensitization testing, which may introduce misclassification and limit distinction between allergic and non-allergic phenotypes. Direct validation studies of the KNHANES physician-diagnosis items are limited. This would most likely attenuate true associations toward the null, although bias in either direction cannot be excluded. In addition, objective biomarkers of nutrient status or inflammatory pathways were not available for inclusion, and residual confounding from lifestyle or environmental factors (e.g., physical activity, alcohol intake, environmental exposures, and supplement use) cannot be excluded. These unmeasured factors may influence both dietary behaviors and allergic disease status, potentially biasing the observed associations. Because many nutrients are consumed together in the same foods, correlations between nutrients are unavoidable in observational dietary analyses. As a result, associations observed in single-nutrient models may partly reflect broader dietary patterns rather than independent nutrient effects. While the present findings were consistent across the primary adjusted models, further stratified or sensitivity analyses may help confirm their robustness in future work. Future studies using dietary pattern analyses or validated dietary indices may better reflect the complexity of diet-allergic disease relationships beyond single-nutrient models, as suggested by recent population-based studies^48,49^. Methods such as principal component analysis or factor analysis could provide complementary insights. Finally, given the large number of statistical tests performed, some associations may represent chance findings despite FDR correction and should be interpreted with caution.

## Conclusion

In conclusion, this nutrient-wide evaluation in Korean adults showed that relationships between nutrient intake and allergic diseases vary by phenotype. Although causal inference is limited by the cross-sectional design, the observed nutrient-wide profiles underscore heterogeneity across asthma, atopic dermatitis, and allergic rhinitis. These findings provide a basis for prioritizing candidate nutrients and broader dietary patterns for validation in future prospective and mechanistic studies.

## Data Availability

The data that support the findings of this study are publicly available from the Korea National Health and Nutrition Examination Survey (KNHANES), provided by the Korea Disease Control and Prevention Agency (KDCA). The datasets are de-identified and can be accessed through the KNHANES website ( [https://knhanes.kdca.go.kr](https:/knhanes.kdca.go.kr) ) in accordance with the data access policies of the KDCA.
